# Facile Fabrication of Hierarchical Structured Anodic Aluminum Oxide Molds for Large-Scale Production of Superhydrophobic Polymer Films

**DOI:** 10.3390/polym16162344

**Published:** 2024-08-19

**Authors:** Athinarayanan Balasankar, Raja Venkatesan, Dae-Yeong Jeong, Tae Hwan Oh, Seong-Cheol Kim, Alexandre A. Vetcher, Subramaniyan Ramasundaram

**Affiliations:** 1 Department of Physics, Gobi Arts and Science College, Gobichettipalayam 638453, India; 2 Nano-Hybrid Technology Research Center, Korea Electrotechnology Research Institute, 9 Beon-gil, 12 Bulmosan-gil, Seongsan-gu, Changwon 51543, Republic of Korea; dyjeong@keri.re.kr; 3 School of Chemical Engineering, Yeungnam University, 280 Daehak-Ro, Gyeongsan 38541, Republic of Korea; taehwanoh@ynu.ac.kr (T.H.O.); sckim07@ynu.ac.kr (S.-C.K.); 4 Nanoeco. Co., Ltd., Technology Start-up Center, Seongju-dong, 10 Jeongiyigil, Seongsan-ku, Changwon 50062, Republic of Korea; 5 Institute of Biochemical Technology and Nanotechnology, Peoples’ Friendship University of Russia n.a. P. Lumumba (RUDN), 6 Miklukho-Maklaya St., 117198 Moscow, Russia; avetcher@gmail.com

**Keywords:** superhydrophobic, polymeric films, anodic aluminum oxide, phosphoric acid anodizing, hierarchically porous, UV lithography

## Abstract

Anodized aluminum oxide (AAO) molds were used for the production of large-area and inexpensive superhydrophobic polymer films. A controlled anodization methodology was developed for the fabrication of hierarchical micro–nanoporous (HMN) AAO imprint molds (HMN-AAO), where phosphoric acid was used as both an electrolyte and a widening agent. Heat generated upon repetitive high-voltage (195 V) anodization steps is effectively dissipated by establishing a cooling channel. On the HMN-AAO, within the hemispherical micropores, arrays of hexagonal nanopores are formed. The diameter and depth of the micro- and nanopores are 18/8 and 0.3/1.25 µm, respectively. The gradual removal of micropatterns during etching in both the vertical and horizontal directions is crucial for fabricating HMN-AAO with a high aspect ratio. HMN-AAO rendered polycarbonate (PC) and polymethyl methacrylate (PMMA) films with respective water contact angles (WCAs) of 153° and 151°, respectively. The increase in the WCA is 80% for PC (85°) and 89% for PMMA (80°). On the PC and PMMA films, mechanically robust arrays of nanopillars are observed within the hemispherical micropillars. The micro–nanopillars on these polymer films are mechanically robust and durable. Regular nanoporous AAO molds resulted in only a hydrophobic polymer film (WCA = 113–118°). Collectively, the phosphoric acid-based controlled anodization strategy can be effectively utilized for the manufacturing of HMN-AAO molds and roll-to-roll production of durable superhydrophobic surfaces.

## 1. Introduction

Superhydrophobic surfaces in which the water contact angle is >150° have been intensively studied over a decade due to the importance of their industrial applications [1,2,3,4,5,6,7,8,9,10]. Superhydrophobic surfaces can be prepared either by tailoring the surface morphology [11,12] or by coating the appropriate hydrophobic materials [13]. A typical example of superhydrophobic surfaces is a lotus leaf, where an array of nanopillars is organized in nipple-like protruded microstructures [14,15]. In order to mimic the surface morphology of a superhydrophobic lotus leaf, surfaces with micro- and nanopatterns are formed using various fabrication techniques such as soft lithography [16], chemical etching [17], solvent-assisted UV-lasering [18], 3D printing [19], and electro-brush plating [20]. One of the ways to mass-produce such superhydrophobic surfaces is replication of the surface morphology of molds by rapid imprinting methods such as thermal imprinting [21]. Specifically, the most important prerequisite for mass production of superhydrophobic polymeric films is the large-scale and cost-effective fabrication of imprint molds with tailored micro- and nanoporous patterns.

Metallic molds possess higher wear resistance and a longer life than molds based on rubber, wood, polymers, or plastics. Metallic molds have been fabricated by various methods, including thermal nanoimprint lithography [22], focused ion beam, femto-second laser, eximer laser, and dicing techniques [23], advanced edge lithography [24], and anodization [25]. Anodization is known as one of the most simple, cheap, and reproducible methods to fabricate imprinting molds. Due to valve metal characteristics, the formed AAO works as a strong protective layer against natural corrosion and enhances the life of the mold surface [26,27,28]. The surface of AAO imprinting molds is usually designed with a variety of organized hexagonal arrays of columnar nanopore channels. The large number of air pockets trapped between water droplets and gaps between pillars on the surface is believed to be responsible for the superhydrophobicity of the surface [29]. The self-ordering is mainly influenced by the applied anodization voltage as well as the purity of the aluminum used [30,31]. The inter-pore distance varies between tens and hundreds of nm in size, depending on the anodization regime [31,32,33]. Besides that, as the pores grow in a direction perpendicular to the anodized surface, a homogenous and smooth surface is required to form a self-ordered array of well-defined nanopores. Also, self-ordering is significantly promoted by performing a two-step anodization [31]. In order to form a relatively thick AAO layer, first the aluminum plate was anodized for a relatively long duration, and then the AAO layer was etched out to leave an array of concave nanoseeds on the aluminum surface [34]. Anodization is repeated multiple times so that the nanopores grow in a well-ordered way at the center of the nanoseeds.

Anodization of aluminum has been widely carried out in three different acidic electrolytes with various voltages: 0.3 M sulfuric at 25 V, 0.3 M oxalic at 40 V, and 0.1 M phosphoric at 195 V [35,36,37]. Self-ordering regimes were limited at higher voltages for oxalic acid and sulfuric acid anodizing. Thus, producing AAO molds with arrays of sub-micron pores requires tedious process control. As a self-ordering regime is achieved at a high voltage in phosphoric acid, it is feasible to produce a sub-micron porous AAO mold with an inter-pore distance of 500 nm. Therefore, a phosphoric acid electrolyte in a 195 V anodizing regime was found to be an appropriate option. Moreover, aluminum is more stable against phosphoric acid than sulfuric acid. Phosphoric acid can also be used as both an electrolyte and a widening agent. However, because of the application of high voltage (195 V), the control of heat dissipation in the phosphoric acid-based anodization process is considered a highly crucial step. Poor heat dissipation causes a catastrophic increase in induced current, which leads to the burning of oxide films and the collapse of the mold surface. The other important issue pertaining to AAO-based imprint molds is the requirement for strong and highly expensive etchants, such as aluminum etchant type A. These kinds of strong etchants are known to be damaging to the interface between the photoresist (PR) used in the patterning process and the aluminum and cause both horizontal and vertical etching. So, it is very difficult to obtain microporous patterns with the required depth. The aforementioned issues must be addressed to obtain low-cost imprint molds as well as the mass production of polymeric superhydrophobic films.

From Table 1, existing methods are not adequate for mass production by industries due to cost, complex procedures, time consumption, and long life. These drawbacks are overcome by the present work. In the present investigation, efforts were made to prepare an AAO imprint mold with a hierarchical micro–nanoporous structure. To address the heat dissipation issue, a phosphoric acid-based anodizing system installed with separate sample- and electrolyte-cooling channels was proposed. A controlled, four-step anodizing–etching procedure was used. Phosphoric acid was used as an electrolyte for anodization and as a widening agent for etching. The fabricated hierarchical micro–nanoporous AAO imprint mold (HMN-AAO) was used to produce superhydrophobic poly(carbonate) (PC) and poly(methyl methacrylate) (PMMA) films using thermal nanoimprint lithography (NIL). For comparison, a nanoporous AAO imprint mold (N-AAO) was also prepared. The surface morphologies of the AAO imprint mold and superhydrophobic polymer films were studied in detail by a field emission scanning electron microscope (FE-SEM). The wetting behavior of the polymeric films was studied using contact angle goniometry.

## 2. Materials and Methods

### 2.1. Fabrication of Nanoporous AAO Imprinting Molds

**Figure 1:**
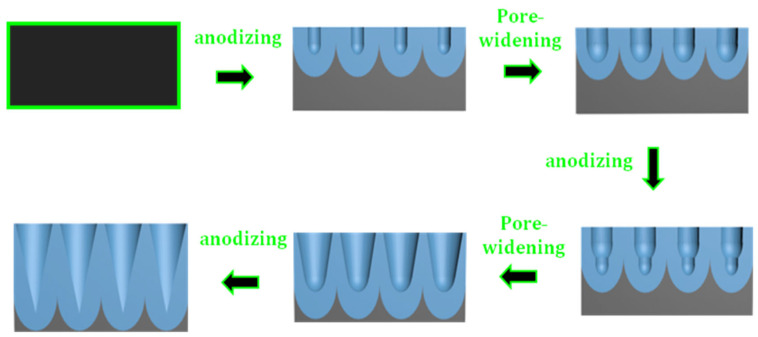
Schematic of the process used for fabricating an AAO imprinting mold with an array of conical nanopores.

Figure 1 shows a schematic of the whole process of fabricating an AAO imprinting mold with an array of conical nanopores. The nanoporous AAO imprinting molds were fabricated using a four-step anodization and etching technique: First, the electro-polished aluminum plate was anodized at −1.2 °C by applying 195 V for 10 h in a 0.1 M phosphoric acid electrolyte. The anodization was performed in a 2 L double-walled glass beaker under vigorous stirring. A constant power supply (OPS 603, ODA Technologies, Incheon, Republic of Korea) was used as a DC power source. During the anodizing process, the current induced was measured and monitored using a Keithley−2100 digital multi-meter interfaced with LabVIEW software. The diameter of the anodized surface was 4 cm. The AAO surface was etched in a mixed solution of 0.19 M chromic acid and 0.1 M phosphoric acid solutions for 60 min at 60 °C. The resultant aluminum surface, which had a highly ordered array of nano-sized concave seeds, was re-anodized for 15 min. The AAO surface formed by the second anodization was etched for 60 min in 0.1 M phosphoric acid at 30 °C. The etched AAO surface was subjected to a third anodization for 15 min and etched for 60 min. Finally, the etched AAO surface was anodized for a fourth time in a series for 15 min and etched for 60 min. Then, the AAO specimen was cleaned with ethanol and dried at 60 °C for 15 min. Except for time duration, in the first to fourth anodization processes, all conditions were the same. The conditions followed for the third and fourth etchings were identical to those of the second etching.

### 2.2. Fabrication of Hierarchical Micro–Nanoporous Imprinting Molds

The HMN-AAO imprinting molds were fabricated on an electro-polished aluminum plate via anodizing, followed by UV lithography. First, the photo resist (PR) (AZ5214E, AZ Electronic Materials, Charlotte, NC 28269, USA) was spin coated on the electro-polished aluminum plate at a spin speed of 4000 rpm for 30 s using a SPIN−1200D (Midas system, Daejeon, Republic of Korea) spin coater. The average thickness of the spin-coated PR film was 2.5 µm. The thickness was measured by a cross-section analysis using a field emission scanning electron microscope (FE-SEM, Hitachi FE-SEM S4800, Tokyo, Japan). The PR-coated specimen was soft-baked at 110 °C for 120 s, covered with a photomask, and exposed to UV light for 100 s using a UV light lithography machine (LABSYS LIT-2000, NEXTRON, Busan, Korea). A negative-type photomask used in the experiment had hexagonally arranged circles (8 µm in diameter) with an inter-circle distance of 24 µm. After UV lithography, the specimen was dried in an oven at 110 °C for 2 min, cooled down to room temperature for 15 min, and developed for 120 s in a developer solution (AMF300, AZ Electronic Materials, Charlotte, NC 28269, USA). The specimen covered with a micropatterned PR layer was hard-baked at 110 °C for 20 min and then anodized at 195 V for 10 h in a 0.1 M phosphoric acid electrolyte. The anodizing temperature was −1.2 °C. Then, the anodized surface was etched with a mixture of 0.19 M chromic acid and 0.1 M phosphoric acid aqueous solutions at 60 °C for 60 min. The etched aluminum plate was subjected to a second anodizing process in a 0.1 M phosphoric acid electrolyte (195 V for 10 min) at −1.2 °C. After the second anodizing, the widening process was performed by immersing the anodized surface in 0.1 M phosphoric acid. Then, the second anodizing and widening processes were identically repeated two times. After electropolishing, successive anodizing, and etching processes, the surface was cleaned by immersing in DI water, ethanol, and acetone in a series for a min and dried at 30 °C for 5 min.

### 2.3. Fabrication of Polymeric Replica Films

First, to remove the oxygen adsorbed on the pore wall surfaces, the fabricated HMN-AAO molds were cleaned using a 1:1 weight/weight mixture of hydrogen peroxide and sulfuric acid for an hour at room temperature (RT). Then, the mold was rinsed with water and ethanol and dried at 60 °C for 20 min. The dried mold was lubricated by immersing in toluene mixed with trichloro (octadecyl)-silane (2 drops/20 g toluene) for 15 h at RT. Then, the mold was rinsed with ethanol and dried at 120 °C for 2 h. The polymeric substrates to be imprinted (PC and PMMA) were also cleaned by sonicating with methanol. The above-cleaned mold and polymeric substrates were mounted on the nanoimprinting lithography (NIL) machine (model no. EITRE^®^ 6, OBDUCAT CO., Nytänkargatan 4, SE-223 63 Lund, Sweden). The mold mounted on the NIL machine was heated up to 130 to 170 °C (glass transition temperature range of polymeric substrates). Then, the pattern on the mold was imprinted on the polymeric substrates by applying a pressure of 30 bars for 5 min. Then, the replica films were detached from the mold, cooled down to room temperature for 15 min, cleaned by using DI water, and dried at 50 °C.

### 2.4. Characterization

The surface morphologies of the N-AAO and HMN-AAO molds and the PC and PMMA replica films were examined using a field emission scanning electron microscope (FE-SEM, Hitachi FE-SEM S4800) operated at 10 kV. The specimens were coated with a layer of osmium by using an osmium plasma coating machine before the FE-SEM observations. The water contact angles (WCAs) of the replica film surfaces were measured at RT using a Phoenix300 contact angle/interface system (Surface Electro Optics Co., Ltd., Suwon, Republic of Korea). They were measured at three different locations on each replica film, and the average values were reported. The volume of the water droplet placed on the surfaces of the replica films was 5 µL.

## 3. Results and Discussion

**Figure 2:**
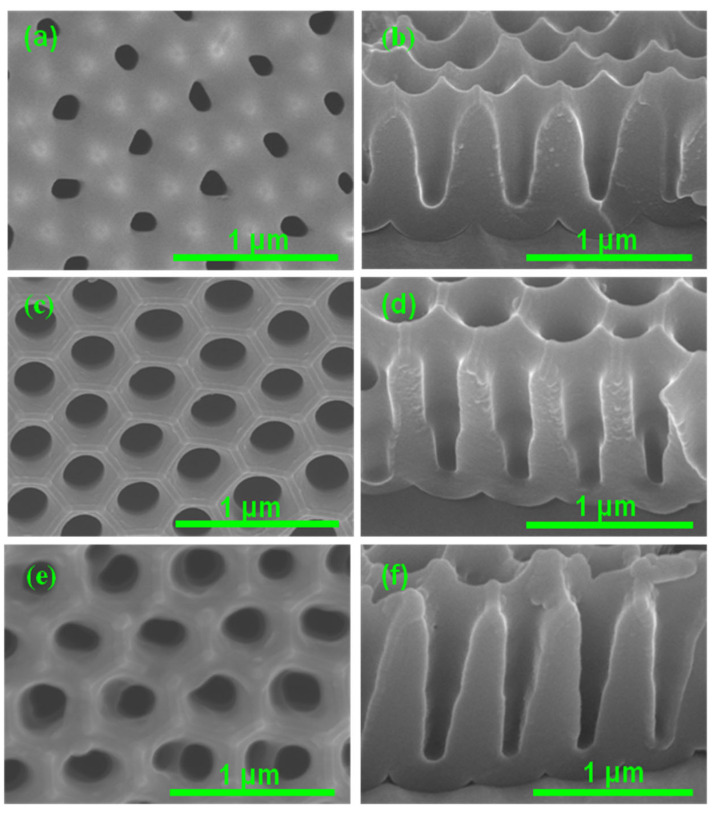
FE-SEM images showing the changes in pore morphology on the top surface (**a**,**c**,**e**) and the cross-section (**b**,**d**,**f**) of AAO templates during the N-AAO process involving multiple anodization and wet-etching steps. The images (**a**,**b**), (**c**,**d**), and (**e**,**f**) were taken after the 2nd, 3rd, and 4th repetitions, respectively.

A conical-shaped nanoporous structure of the AAO imprint mold is essential to increasing the air interface between the surface and water and also to ensuring smooth surface finishing. An increase in the air interface increases the WCA [43]. A smooth surface resulted from an array of strong pillars formed out of a conical-shaped nonporous structure, which facilitates the easy recovery of the replica film from the imprint mold. So, to check the feasibility of our strategy of controlled anodization using a phosphoric acid electrolyte, the first N-AAO was prepared. Figure 2a–f depicts the FE-SEM images of a template obtained as a function of the increase in the number of anodization and subsequent wet etching (widening) steps during the N-AAO process. The latter was performed to increase the pore diameter as well as induce a change in pore shape [43]. In Figure 2, the surface morphology images are organized on the left (Figure 2a,c,e), and the respective cross-sectional images (Figure 2b,d,f) are provided on the right. After the second anodization−widening step, the circular and random pores (206 nm in diameter) were formed on the surface. Notably, these pores were surrounded by a hexagonal array of seeds, formed at the bottom of the barrier layer just after the first anodization−etching step.

**Figure 3:**
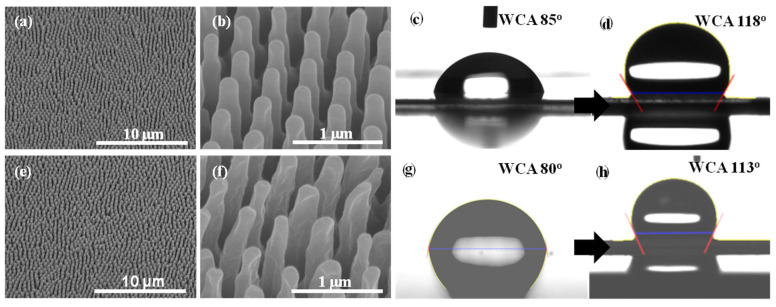
FE-SEM images showing the surface morphology of PC (**a**,**b**) and PMMA (**e**,**f**) films replicated using an N-AAO mold using the thermal imprinting method. (**b**) and (**f**) are the enlarged images of (**a**) and (**e**), respectively. The images (**c**,**d**) and (**g**,**h**) are photographs of water droplets taken on the surfaces of PC and PMMA films, respectively. The images (**c**,**g**) and (**d**,**h**) are WCAs of pristine and nanopillared PC and PMMA replica films.

Figure 3 shows the FE-SEM images of PC (a and b) and PMMA (e and f) replica films prepared by thermal imprinting of N-AAO. The WCA images of pristine and replica films are shown in Figure 3c,g and Figure 3d,h, respectively. In the FE-SEM images with 10 µm resolution, the surface of the replica film was filled with uniformly arranged pillars (top diameter = 154 nm). The conical shape of the pillars was clearly visible in the respective images with 1 µm resolution. The inter-pillar distance was 550 nm, the same as the inter-pore distance noticed in the AAO imprint mold. The pillars appeared intact, and there was no sign of damage. The presence of structurally intact pillars confirmed that the conically shaped nanopores in the AAO mold facilitated the easy separation of the replica film after imprinting without any damage. The WCAs of the PC (Figure 3d) and PMMA (Figure 2h) replica films were 118° and 113°, respectively. After imprinting with N-AAO, the surface of the polymer films was turned from hydrophilic to hydrophobic. The WCA of the pristine PC is 85° (Figure 3c), and it is 80° (Figure 3g) for pristine PMMA. The surface morphology and WCA of the PC and PMMA replica films proved the usability of N-AAO prepared by the proposed 0.1 M phosphoric acid electrolyte-based controlled anodization method for the large-scale production of hydrophobic polymer films.

The cross-sectional view confirmed the formation of conical pores with a depth of 536 nm. Well-ordered hexagonal pores with a diameter of 220 nm were seen after the third anodization−widening, and the depth of the conical pores was 721 nm. After the fourth anodization, the diameter of the pore was further increased to 227 nm, and the pore depth became 1030 nm. With respect to the increase in the number of anodization steps, the pore entrance and the pore diameter proved to be gradually increased. The depth-to-diameter ratio (aspect ratio) of the pores was also increased with the increase in the number of anodization steps; it was 2.77, 3.27, and 4.53 after the second, third, and fourth anodizations, respectively. It is important to emphasize that the increase in pore size, depth, and depth-to-diameter ratio with respect to the increase in the number of anodization steps did not collapse the pore walls. The inter-pore distance (550 nm) also remained unaltered after three repetitive anodization and widening processes. The above results proved that the proposed strategy of controlled anodization with a 0.1 M phosphoric acid electrolyte was useful for preparing an AAO mold with a uniform surface and conical pores.

**Figure 4:**
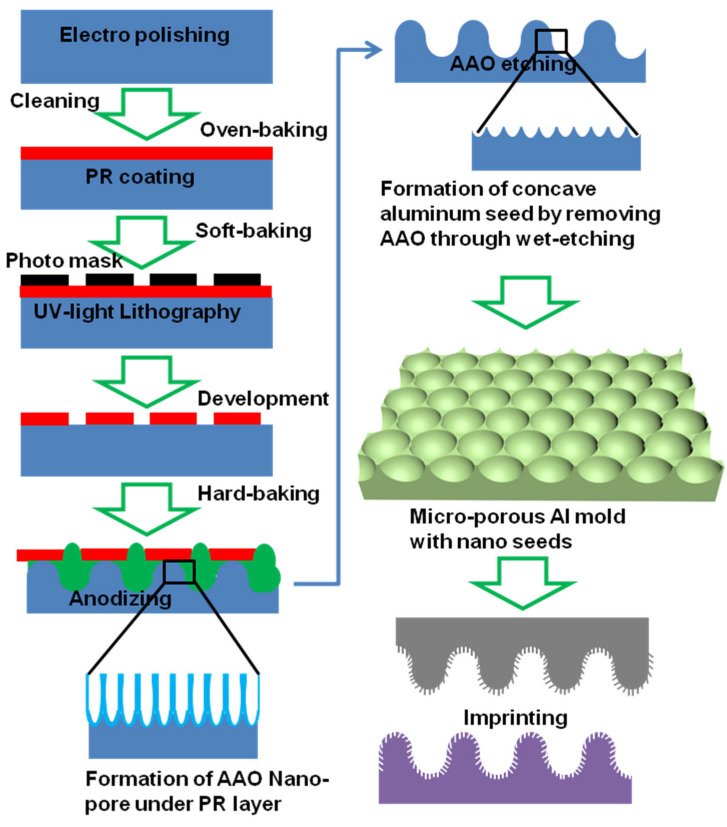
Schematic of the process used for fabricating an HMN-AAO imprint mold using the controlled anodization method.

Figure 4 depicts the schematic of the procedure followed for preparing HMN-AAO by the combination of UV lithography and a controlled anodization strategy optimized for preparing N-AAO. UV lithography is one of the conventional methods used for micropatterning. The shortcomings, such as the need for expensive chemical etching and difficulties in forming high-depth microporous patterns, restrict the use of UV lithography for preparing cost-effective AAO imprint molds. Therefore, to make high-depth microporous arrays, the fabrication process was modified as follows: The aluminum surface covered with the PR layer, micropatterned using UV lithography, was anodized. In order to form the arrays of hexagonal nanoseeds and micropores, the formed AAO was etched by a mixture of phosphoric acid and chromic acid. Then, the surface was re-anodized and etched repeatedly to make conical nanopores on the nanoseeds. As a result, AAO molds with hierarchical structures consisting of arrays of nanopores and higher-depth micropores could be obtained.

**Figure 5:**
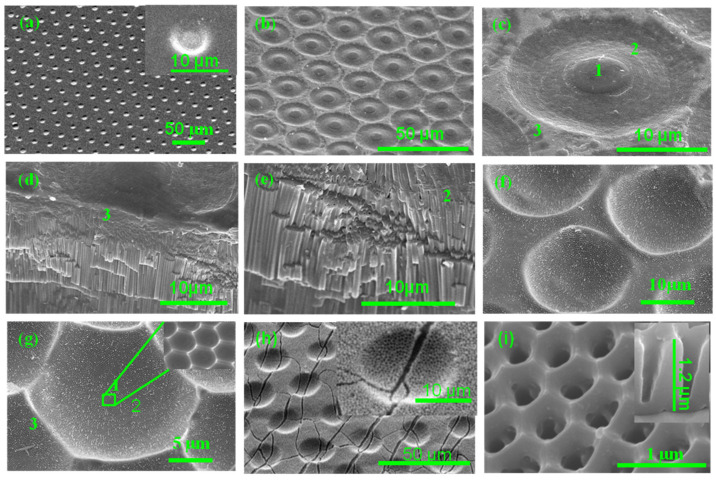
FE-SEM images of a template at various stages of the HMN-AAO fabrication process. (**a**) Micropatterned template after the UV lithography; (**b**,**c**) after anodization in a phosphoric acid electrolyte for 10 h; (**d**) a 45° tilted image of an accidentally cleaved portion near zone 3 in (**c**); (**e**) a 45° tilted image of an intentionally cracked portion near zone 2 in (**c**); (**f**) after etching of the AAO layer formed during the anodization; (**g**) enlarged images of (**f**) showing an array of aluminum seeds in sub-micron size; (**h**,**i**) surface of the HMN-AAO imprint mold after bending.

The changes in the morphology of the aluminum template during the various steps of the HMN-AAO fabrication process (Figure 4) are shown in Figure 5. Uniform micropatterns of PR (MPPR) can be seen on the micropatterned aluminum surface subjected to UV lithography (Figure 5a). The inter-distance between neighboring circles was 24 µm. The average diameter of the single circle was estimated to be 6.7 µm (inset of Figure 5a). The aluminum surface with the MPPR arrays was anodized at 195 V in a phosphoric acid electrolyte for 10 h. In conventional anodization, the nanopore channels used to be straight and uniform. In contrast, after anodizing, both declined, and straight nanopore channels were formed (Figure 5b). For better understanding, in Figure 5c, the straight pore channels, declined nanopore channels, and the boundary are marked as regions 1, 2, and 3, respectively. The interface area between regions 2 and 3 was covered by a PR layer along with nanoporous AAO. This interfacial PR layer acted as a sealant and prevented the diffusion of the electrolyte to the aluminum. The 45° titled cross-sectional view of the declined pore channel (Figure 5d) and boundary (Figure 5e) discerns that the growth rate of the straight pore channel is higher than the declined pore channel. The formation of the straight and declined pore channels during anodization can be explained using the stability of the binding interface between the PR and aluminum and the electrolyte diffusion. In conventional anodization, numerous closely packed pores are formed, and there is no free space in the horizontal direction. Thus, due to the constraints posed by inter-pore repulsive forces, the formed pores grow vertically at the center of the circles as straight channels. In the controlled anodization used here, only in the region of the straight pore channel do AAO pores start to grow up in directions perpendicular to the aluminum surface. Growth in other regions is restricted due to strong binding between the PR layer and aluminum surfaces. However, AAO pores in the vicinity of the circle edges start to grow gradually in all possible directions into aluminum under the PR layer (region 2) because there are no constraints in the radial (horizontal) direction. As a result, the pore channels start to bend to the region under the PR layer. Due to bending and a 40% volume expansion caused by AAO formation [44] at the edges of the circle, the crevices occur at the interface of the PR and aluminum. The electrolyte starts to penetrate through these crevices, and the pores under MPPR grow in both vertical and horizontal directions simultaneously. The growth of both the horizontal and vertical pores continued until the point at which they deviated from the MPPR circles. These points of deviation appeared like barrier layers between the pores grown in the vertical and horizontal directions (Figure 5d,e).

To further obtain insights about the controlled anodization process, the mechanism behind the formation of deep micropores (Figure 5b–e) must be explained. The anodized layer thickness in region 1 gradually decreases when approaching region 3. As a result of a 40% volume expansion during the anodization [44] and a constraint posed by the PR layer, the edges were suppressed and region 1 was protruded (Figure 5c). The height of region 1 appeared to be 4 µm larger than that of region 3. Therefore, the lengths of the straight (vertical) pore channel were deeper (longer) than the bent or declined (horizontal) pore channel. When the entire anodized layer etched out, an array of pores with a depth almost equal to their radius was formed. The PR layer was predicted to remain up to half of the (4–5.3 h) total anodization process (10 h). In the typical aluminum etching, within an hour, the PR layer was usually removed by the aluminum etchant [43]. Due to that, the pores formed therein were not deeper. The average diameter of the micropores in Figure 5g was estimated to be ~18.0 μm. The depth of the micropores in Figure 5g was estimated to be 8.0 μm. The smaller depth of the micropores in region 2 (Figure 5f,g) than their diameter is attributed to the detachment of the PR layer during the anodization. Thus, the shape of the microporous appears concave. Also, it can be inferred that the initial anodization period is crucial for determining the pore depth. The inset in Figure 5g exhibited the formation of a uniform array of hexagonal nanoseeds inside the micropores.

Figure 5h,i illustrates the formation of the hierarchical micro-/nanoporous structure upon completion of fourth anodization and accompanying the widening process. The cracks seen on these images were formed due to folding of the sample prepared for effective observation of the cross-section and top surface. The array of the AAO micropores can be seen in Figure 5h. Due to the growth of the nanopores in the radial direction (inset of Figure 5h), the diameter (17.5 µm) of these AAO micropores was found to be less than the diameter (18.4 µm) of the microporous aluminum with the nanoseed (Figure 5f,g). Due to the cracks, the inter-pore distance appears to be larger than the microporous aluminum with the nanoseed. As seen in the morphology of a single AAO micropore, shown in the inset of Figure 5h, the arrays of nanopores were distributed on both the boundary and micropore region. As seen in Figure 5i and inset therein, these nanopores were hexagonal in shape with a diameter and depth of 300 nm and 1.2 µm, respectively. The obtained HMN-AAO was used for preparing the polymeric replica film.

**Figure 6:**
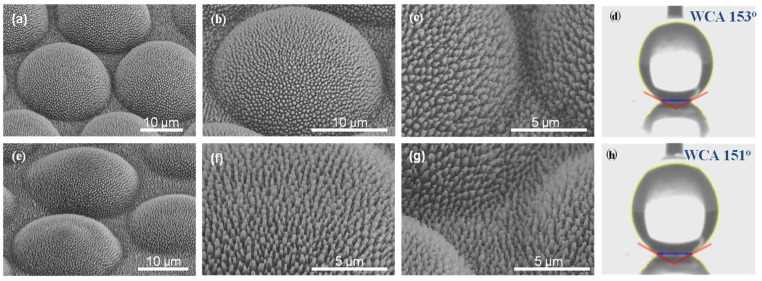
FE-SEM images of PC (**a**–**c**) and PMMA (**e**–**g**) replica films prepared using an HMN-AAO imprinting mold. The magnification was increased in the order of (**a**–**c**), and (**e**–**g**), respectively. The images (**d**) and (**h**) are WCA photographs of PC and PMMA replica films, respectively.

Figure 6 shows the FE-SEM images of PC (a–c) and PMMA (e–g) films replicated using the HMN-AAO. The WCA photographs of the resultant PC and PMMA replica films are shown in Figure 6d,h. FE-SEM images with 10 and 5 µm resolution were provided to effectively explain the micro- and nanostructure on the replica films. Upon replication, the well-organized micropillars comprising the array nanopillars were created on both the PC and PMMA surfaces. The diameters of the micro and nanopillars were 21.42 and 0.306 µm, respectively. The absence of breakages discerns that the formed micro- and nanopillars possess sufficient mechanical stability to withstand forces exerted on the replica films while imprinting, especially during the separation of the mold after imprinting. When compared with replica films prepared using N-AAO (Figure 2e,f), the HMN-AAO led to an increase in the WCAs of PC (153°) and PMMA (151°), respectively. In other words, the N-AAO mold enhanced the hydrophobicity, and HMN-AAO imparted the superhydrophobicity. The significant increase in the WCA was attributed to the presence of an array of nanopillars within the micropillar structures. Specifically, this hierarchal micro–nanopore structure increases the effective number of air pockets trapped between water droplets and gaps between micropillars. The increase in the number of air pockets increased the WCA. Overall, the morphology and WCA proved that HMN-AAO fabricated by the controlled anodization process is suitable for the mass production of superhydrophobic polymeric films using the thermal nanoimprinting method. This strategy is more advantageous than the previous methods reported for the fabrication of micro-/nanopillared superhydrophobic PDMSs [45], where silicon nanocrystals were pasted on a micropatterned PDMS obtained by a two-step soft-lithography process. Also, due to the weaker binding of pasted nanocrystals on the micropatterned surface, the superhydrophobic property may decay over a short span of time and use. The mechanically robust micro–nanopillars created on the replica films by imprinting HMN-AAO are considered permanent and durable over a longer time.

## 4. Conclusions

The low-cost and considerably safer phosphoric acid electrolyte-based controlled anodization method was established for the fabrication of HMN-AAO imprint molds. An HMN-AAO mold with the dimension of 100 × 100 mm^2^ was fabricated by phosphoric acid anodizing at 195 V. The relevant thermodynamic parameters were controlled and optimized by establishing a special cooling system. The total production time and cost were minimized by using phosphoric acid as both the electrolyte and widening agent. The morphology of the HNM-AAO mold observed by FE-SEM confirmed the formation of arrays of hexagonal nanopores within the hemispherical micropore structures with a high aspect ratio. Both the micro- and nanopore structures were uniform and had distinct boundaries. The superhydrophobic PC and PMMA films exhibiting WCAs of 153° and 151°, respectively, were prepared by thermally imprinting the HMN-AAO mold. Imprinting of the HMN-AAO mold resulted in the formation of mechanically robust hierarchical micro–nanopillars on the surface of the polymeric films, whereas thermal imprinting of the N-AAO mold only increased the WCA of the hydrophilic PC (85°) and PMMA (80°) to hydrophobic PC (118°) and PMMA (113°). The above-established phosphoric acid electrolyte-based controlled anodization strategy was proved to have the potential for the large-scale commercial production of HMN-AAO imprint molds as well as durable superhydrophobic polymeric films.

## Figures and Tables

**Table 1:** Comparison table for fabrication of large-scale superhydrophobic films.

Methodology	Advantages	Challenges	References
Electrochemical deposition method	Even on a large scale, it is very easy to control the thickness of the deposition layer	This will not work in non-conductive materials	[38]
Spin coating methods	Minimum curing time	Poor binding energy between the substrates and polymers	[39]
Chemical vapor deposition method	Portable, precise thickness control	Very expensive and not adequate for mass production	[40]
Chemical etching method	Stable due to single-material work as a substrate	Expensive, time-consuming, and poor mechanical strength	[41]
Spray coating method	Simple operation procedure	Consumed too many coating materials and did not have strong binding	[42]
Combined methodology of UV lithography and anodization for master mold fabrication	Very cheap, simple, high throughput, mechanically stable	Stamping only possible for flexible polymers	Current work

## Data Availability

Upon reasonable request, the data supporting this investigation are available from the corresponding authors.

## References

[1] Shibuichi S., Yamamoto T., Onda T., Tsujii K. (1998). Super Water and Oil-Repellent Surfaces Resulting from Fractal Structure. J. Colloid Interface Sci..

[2] Nakajima A., Hashimoto K., Watanabe T., Takai K., Yamauchi G., Fujishima A. (2000). Transparent Superhydrophobic Thin Films with Self-Cleaning Properties. Langmuir.

[3] Wang B., Liang W.X., Guo Z.G., Liu W.M. (2015). Biomimetic super-lyophobic and super-lyophilic materials applied for oil/water separation: A new strategy beyond nature. Chem. Soc. Rev..

[4] Valipour N.M., Birjandi F.C., Sargolzaei J. (2014). Super-non-wettable surfaces: A review. Colloids Surface A.

[5] Guo Z.G., Liu W.M., Su B.L. (2011). Superhydrophobic Surfaces: From Natural to Biomimetic to Functional. J. Colloid Interface Sci..

[6] Lv J.Y., Song Y.L., Jiang L., Wang J.J. (2014). Bio-Inspired Strategies for Anti-Icing. ACS Nano.

[7] Jeevahan J., Chandrasekaran M., Joseph G.B., Durairaj R., Mageshwaran G. (2018). Superhydrophobic surfaces: A review on fundamentals, applications, and challenges. J. Coat. Technol. Res..

[8] Das S., Kumar S., Samal S.K., Mohanty S., Nayak S.K. (2018). A review on superhydrophobic polymer nanocoatings: Recent development, application. Ind. Eng. Chem. Res..

[9] Shome A., Das A., Borbora A., Dhar M., Manna U. (2022). Role of chemistry in bio-inspired liquid wettability. Chem. Soc. Rev..

[10] Hu Z., Chu F., Shan H., Wu X., Dong Z., Wang R. (2023). Understanding and Utilizing Droplet Impact on Superhydrophobic Surfaces: Phenomena, Mechanisms, Regulations, Applications, and Beyond. Adv. Mater..

[11] Gou X., Guo Z. (2019). Surface topographies of biomimetic superamphiphobic materials: Design criteria, fabrication and performance. Adv. Colloid Interface Sci..

[12] Ma M.L., Hill R.M., Lowery J.L., Fridrikh S.V., Rutledge G.C. (2005). Electrospun Poly(Styrene-Block-Dimethylsiloxane) Block Copolymer Fibers Exhibiting Superhydrophobicity. Langmuir.

[13] Wang H., Dai D., Wu X. (2008). Fabrication of superhydrophobic surfaces on aluminum. Appl. Surf. Sci..

[14] Wang H.X., Fang J., Cheng T., Ding J., Qu L.T., Dai L.M., Wang X.G., Lin T. (2008). One-Step Coating of Fluoro- Containing Silica Nanoparticles for Universal Generation of Surface Superhydrophobicity. Chem. Commun..

[15] Yu S., Guo Z.G., Liu W.M. (2015). Biomimetic transparent and superhydrophobic coatings: From nature and beyond nature. Chem. Commun..

[16] Wang F., Li S., Wang L. (2017). Fabrication of artificial super-hydrophobic lotus-leaf-like bamboo surfaces through soft lithography. Colloid Surface A.

[17] Shi T., Xue S., Ma X., Peng H., Du J., Zheng B., Xia Z. (2021). Fabrication of superhydrophobic micro-nanostructured aluminum alloy surface via a cost-effective processing using an ultra-low concentration of fluoroalkylsilane. Appl. Phys. A.

[18] Zhang S., Jiang Q., Xu Y., Guo C.F., Wu Z. (2020). Facile Fabrication of Self-Similar Hierarchical Micro-Nano Structures for Multifunctional Surfaces via Solvent-Assisted UV-Lasering. Micromachines.

[19] Wei Y., Hu Y., Li M., Li D. (2021). Fabrication of Sr-functionalized micro/nano-hierarchical structure ceramic coatings on 3D printing titanium. Surf. Eng..

[20] Liu H., Wang X., Ji H. (2014). Fabrication of lotus-leaf-like superhydrophobic surfaces via Ni-based nano-composite electro-brush plating. Appl. Surf. Sci..

[21] Brock L., Sheng J. (2020). Robust fabrication of polymeric nanowire with anodic aluminum oxide templates. Micromachines.

[22] Kwon S., Kim Y., Lim H., Kim J., Choi K., Lee J., Kim G. (2020). Fabrication of a Metal Roller Mold with Nanoimprinted Pattern Using Thermal Nanoimprint Lithography. Sci. Adv. Mater..

[23] Youn W., Takahashi M., Goto H., Maeda R. (2007). Fabrication of micro-mold for glass embossing using focused ion beam, femto-second laser, eximer laser and dicing techniques. J. Mater. Process. Technol..

[24] Sakamoto J., Nishino T., Kawata H., Yasuda M., Hirai Y. (2011). High aspect ratio nano mold fabrication by advanced edge lithography without CVD. Microelectron. Eng..

[25] Lee W., Park S.-J. (2014). Porous anodic aluminum oxide: Anodization and templated synthesis of functional nanostructures. Chem. Rev..

[26] Parvate S., Dixit P., Chattopadhyay S. (2020). Superhydrophobic Surfaces: Insights from Theory and Experiment. J. Phys. Chem. B.

[27] Darmanin T., Guittard F. (2015). Superhydrophobic and superoleophobic properties in nature. Mater. Today.

[28] Jeong C., Choi C.-H. (2012). Single-step direct fabrication of pillar-on-pore hybrid nanostructures in anodizing aluminum for superior superhydrophobic sufficiency. ACS Appl. Mater. Interfaces.

[29] Bravo J., Zhai L., Wu Z., Cohen R.-E., Rubner M.-F. (2007). Transparent superhydrophobic films based on silica nanoparticles. Langmuir.

[30] Michalska-Domańska M., Norek M., Stępniowski W.J., Budner B. (2013). Fabrication of high quality anodic aluminum oxide (AAO) on low purity aluminum—A comparative study with the AAO produced on high purity aluminum. Electrochim. Acta.

[31] Lee W., Ji R., Gösele U., Nielsch K. (2006). Fast fabrication of long-range ordered porous alumina membranes by hard anodization. Nat. Mater..

[32] Surawathanawises K., Cheng X. (2014). Nanoporous anodic aluminum oxide with a long-range order and tunable cell sizes by phosphoric acid anodization on pre-patterned substrates. Electrochim. Acta.

[33] Masuda H., Fukuda K. (1995). Ordered Metal Nanohole Arrays Made by a Two-Step Replication of Honeycomb Structures of Anodic Alumina. Science.

[34] Kim D., Kim J., Park H.-C., Lee K.-H., Hwang W. (2008). A superhydrophobic dual-scale engineered lotus leaf. J. Micromech. Microeng..

[35] Nielsch K., Choi J., Schwirn K., Wehrspohn R.B., Gosele U. (2002). Self-ordering regimes of porous alumina: The 10% porosity rule. Nano Lett..

[36] Zhao N.Q., Jiang X.X., Shi C.S., Li J.J., Zhao Z.G., Du X.W. (2007). Effects of anodizing conditions on anodic alumina structure. J. Mater. Sci..

[37] Masuda H., Yada K., Osaka A. (1998). A Self-Ordering of Cell Configuration of Anodic Porous Alumina with Large-Size Pores in Phosphoric Acid Solution. Jpn. J. Appl. Phys..

[38] Tian J., Bao J., Li L., Sha J., Duan W., Qiao M., Cui J., Zhang Z. (2023). Facile fabrication of superhydrophobic coatings with superior corrosion resistance on LA103Z alloy by one-step electrochemical synthesis. Surf. Coat. Technol..

[39] Pawar P.G., Xing R., Kambale R.C., Kumar A.M., Liu S., Latthe S.S. (2017). Polystyrene assisted superhydrophobic silica coatings with surface protection and selfcleaning approach. Prog. Org. Coat..

[40] Fu J., Sun Y., Ji Y., Zhang J. (2022). Fabrication of robust ceramic based superhydrophobic coating on aluminum substrate via plasma electrolytic oxidation and chemical vapor deposition methods. J. Mater. Process. Technol..

[41] Sinha A., Gupta M.C. (2021). Microscale patterning of semiconductor c-Si by selective laser-heating induced KOH etching. Semicond. Sci. Technol..

[42] Shen Y., Li K., Chen H., Wu Z., Wang Z. (2021). Superhydrophobic F-SiO2@PDMS composite coatings prepared by a two-step spraying method for the interface erosion mechanism and anti-corrosive applications. Chem. Eng. J..

[43] Athinarayanan B., Jeong D.Y., Kang J.H., Koo B.H. (2015). Fabrication of hydrophobic and anti-reflective polymeric films using anodic aluminum-oxide imprints. J. Korean Phys. Soc..

[44] Pu Y., Hu J.J., Yao T., Li L., Zhao J., Guo Y. (2021). Influence of anodization parameters on film thickness and volume expansion of thick- and large-sized anodic aluminum oxide film. J. Mater. Sci. Mater. Electron..

[45] Gao X., Yan X., Yao X., Xu L., Zhang K., Zhang J., Yang B., Jiang L. (2007). The Dry-Style Antifogging Properties of Mosquito Compound Eyes and Artificial Analogues Prepared by Soft Lithography. Adv. Mater..

